# The Use of Aluminium Alloy after High Plastic Deformation for Joining Riveted Structures

**DOI:** 10.3390/ma15051920

**Published:** 2022-03-04

**Authors:** Bogdan Szturomski, Radosław Kiciński

**Affiliations:** Mechanical and Electrical Engineering Department, Polish Naval Academy, 81-103 Gdynia, Poland; bsztur@gmail.com

**Keywords:** Al7.5Mg aluminium alloy, blunt impact test, static tensile test, dynamic tensile test, FEM simulation, Johnson–Cook material model

## Abstract

This paper presents the results of a static and dynamic tensile test of an Al7.5Mg aluminium alloy taken from round bars made in the technology of hydrostatic extrusion. It is planned to use the Al7.5Mg aluminium alloy for joining riveted structures. Based on the obtained results, the nominal and true characteristics of the Al7.5Mg aluminium alloy, depending on the strain rate in the range from 0 to 2000 s^−1^, were developed. The failure criterion for tension was determined. The material characteristics were approximated by the Johnson–Cook equation, which can be used in CAE (computer-aided engineering) programs to simulate the impact processes. FEM (finite element method) simulation of the impact of the hammer on the part of the riveted aircraft structure was performed. The FEM simulation results were compared with the experimental results on a drop hammer to verify the material model. The following results were obtained: yield strength *R_e_* = 395.3 MPa; strength limit *R_m_* = 523.1 MPa at deformation 0.067; Young’s modulus *E* = 7.9 × 10^4^ MPa. The AL7.5Mg alloy after hydro-extrusion has favourable plastic and strength properties.

## 1. Introduction

There are many aspects to consider when constructing and designing any technical device. Designing machines and devices is based on finding innovative solutions that will make a given structure unique and different from others. However, it is necessary to follow certain construction principles, such as functionality, reliability, ergonomics, and efficiency. Each machine or device should also have a good value for its cost. In the case of warships, an essential factor is a mass above the centre of gravity [1], and in the case of aircraft structures, mass is a general factor.

Currently, the world is looking for solutions to reduce the weight of ships. Numerical and technological methods allow for more accurate calculations, which translates into the thickness of the structural reinforcements. In contrast, modern technologies such as friction welding (FSW) [2] translate into a reduction in the weight of joints. Another approach to weight reduction is using materials with higher specific strength, understood as the mechanical strength of a given material in relation to the specific weight [3]. Aluminium–magnesium alloys can undoubtedly be used for the production of rivets. They can probably also be used in several other machine elements, especially where weight reduction is necessary for aviation and marine structures or underwater biomimetic vehicles [4,5]. Aluminium alloys have many advantages over steel: their density is 2.9 times lower, they do not rust in a marine environment, they are non-magnetic, and they conduct heat perfectly [6].

Unlike in shipbuilding, riveting is still used to connect aircraft structures. This is due to the low mass of the connecting elements that constitute a significant aircraft mass, considering the economies of scale [7].

One of the ideas for reducing the weight of aircraft structures is the use of alloys with increased strength for rivet materials. This will reduce their diameter and weight. Increased strength of aluminium alloys can be obtained using Severe Plastic Deformation (SPD) processes. Such methods include equal channel angular extrusion (ECAE) [8], equal-channel angular hydro-extrusion (ECAH) [9,10], high-pressure torsion (HPT) [11], accumulative roll bonding (ARB) [12], repetitive corrugation and straightening (RCS) [13], and asymmetric rolling (ASR) [14].

## 2. Materials and Methods

In this study, we tested the aluminium–magnesium alloy Al7.5Mg (7.5% magnesium content) after the process of hydrostatic extrusion, with the degree of strengthening equal to $\overset{´}{\varepsilon }=0.86$, intended for the production of rivets for aircraft structures. Hydro-extrusion is one of the many technologies of high plastic deformation, which enables controlled shaping of the microstructure, thus increasing the strength and plastic properties of the material. The degree of strengthening was calculated according to the formula [8,11,15,16,17]:

(1) $$\overset{´}{\varepsilon }=2\cdot \ln\frac{\emptyset_{i}}{\emptyset_{e}}=2\cdot \ln\frac{20\;\mathrm{mm}}{13\;\mathrm{mm}}=0.86$$

where:

$\emptyset_{i}$—initial bar diameter; and

$\emptyset_{e}$—end bar diameter.

The aim of the work is to determine the material characteristics of Al7.5Mg alloys after hydro-extrusion for numerical calculations. The developed characteristics will then be used in FEM programs to determine the strength of riveted structures, such as aircraft plating. Aircraft structures work in challenging conditions. They are also exposed to impacts. At the design stage of an aircraft structure, it is necessary to perform several numerical tests and simulations, including fast-changing processes such as impact. Dynamic material characteristics depending on the strain rate are needed for these simulations. Before performing the FEM simulation, the characteristics of the material should be verified with simple laboratory experiments, which will allow determining their suitability.

**Figure 1:**
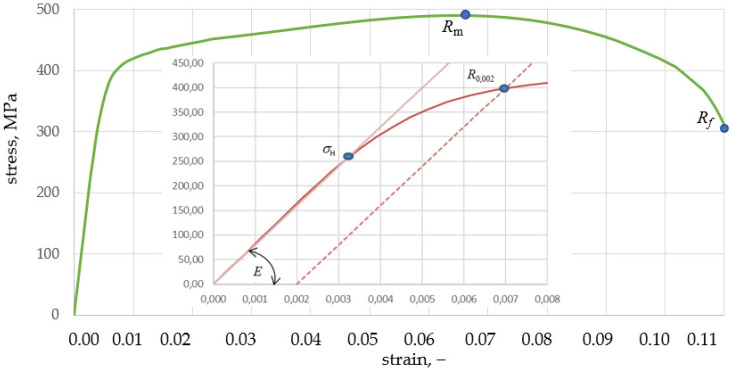
Nominal characteristics of the Al7.5Mg aluminium alloy after hydro-extrusion.

The static tensile test was carried out on the MTS testing machine, on which standard samples with a diameter of 8 mm were stretched in accordance with the EN ISO 6892-1: 2016 standard [18]. The nominal characteristics, determined directly from the machine based on the tensile force and measurement from an extensometer with a working length of 40 mm, are shown in Figure 1.

The nominal sizes are as follows:
Young’s modulus *E* = 7.9 × 10^4^ MPaelastic range of material ($\sigma_{H}=\sigma_{\mathrm{pl}=0}$) *A* = 258 MPa with strain *ε* = 0.0032Yield stress *R*_0.002_ = 392 MPa with strain *ε*_0.002_ = 0.0068Ultimate Strength *R_m_* = 490 MPa with strain *ε_m_* = 0.0653Fracture *R_f_* = 306 MPa with strain *ε_f_* = 0.1103

Using the dependence between the true stresses *σ*_*true*_ and nominal stresses *σ_nom_*, the volume of the stretched sample during stretching is constant, so:

*l*_0_*A*_0_ = *l A(F)* (2)

Formulas (2)–(6) included in Table 1 determine the true and plastic characteristics [19]. Table 1 contains calculations for selected measuring points.

### The Study of Dynamic Mechanical Properties Using a Rotary Hammer

**Figure 2:**
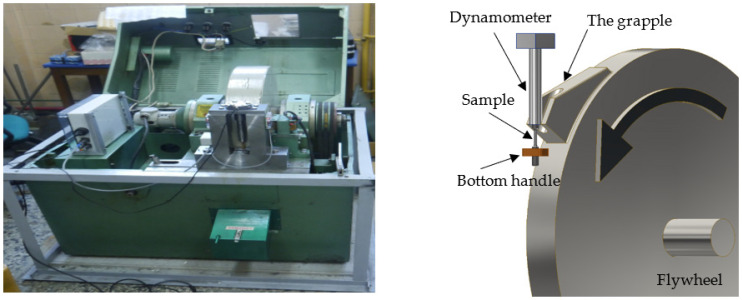
Rotary hammer station (Fundamentals of Technology Laboratory, Polish Naval Academy) and scheme of dynamic tensile test on a rotary hammer.

The Fundamentals of Technology Laboratory of the Naval Academy in Gdynia has a unique stand, a rotary hammer (Figure 2) that enables the dynamic tensile test at speeds in the range of 10 ÷ 50 m/s. A sample length of 20 mm allows the strain rate to be equal to 500 ÷ 2000 s^−1^. In Poland, the Silesian University of Technology still has similar laboratory equipment [20].

The rotary hammer station enables samples to be picked with a strain rate in the range of 0 ÷ 2000 s^−1^. The rate of deformation can be determined from the formula [21]:

(8) $$\dot{\varepsilon }=\frac{d\varepsilon }{dt}=\frac{d}{dt}\left(\frac{\upsilon \cdot t}{l}\right)=\frac{\upsilon }{l}$$

Table 2 shows the results of sample breaking using a rotary hammer. The strain rates in the range of 535–2159 s^−1^ were obtained, for which the values of the strength limit *R_m_* increase to over 631 MPa.

In CAE programs, functions $\sigma_{true}=\sigma_{true}\;\left(\varepsilon_{pl},\dot{\varepsilon },\;\theta \right)$ in the form of a polynomial are used to describe the plastic characteristic depending on the strain rate and temperature. The Johnson–Cook constitutive model is widely used in CAE programs [22]. Given is a pattern:

(9) $$\sigma_{pl}=\left(A+B\varepsilon_{pl}^{n}\right)\left[1+C\ln\left(\frac{\dot{\varepsilon }}{{\dot{\varepsilon }}_{0}}\right)\right]\left[1-{\left(\frac{\theta -\theta_{0}}{\theta_{melt}-\theta_{0}}\right)}^{m}\right]\;$$

where:

*A*—elastic range of the material $\sigma_{pl=0}$*(*$\varepsilon_{pl}\leq \;$0.00002), it is often simplified in form *A = R_e_*;

*B*—hardening parameter;

*n*—hardening exponent;

*C*—strain rate coefficient;

$\varepsilon_{pl}$—true plastic strain;

$\dot{\varepsilon }$—strain rate;

${\dot{\varepsilon }}_{0}$– quasi-static strain rate (0.0001 s^−1^);

*θ*—current material temperature;

*θ*_0_—ambient temperature;

*θ_melt_*—melting temperature; and

*m*—thermal softening exponent.

Parameters *A, B, C, n*, and *m* can be determined in many ways [21,23,24,25] from the transformation of individual members of Equation (9). The results obtained from static and dynamic tests are substituted. We propose the following algorithm. From the nominal or real characteristic (in the proportionality range, both characteristics coincide), the coefficient A is determined: proportionality limit $\sigma_{H}=\sigma_{pl=0}$ for $\varepsilon_{pl}\leq \;$0.00002.

In the first part of Equation (9), there are two unknown coefficients, *B* and *n*, constituting a dependent pair. From the transformation of the first term of Equation (9), we obtain:

(10) $$B=\frac{R_{m,true}-A}{\varepsilon_{m,pl}^{n}}\;$$

The pair of coefficients *B* and *n* must satisfy Equation (9) at the point *R_e,true_* and *R_m,true_*:

(11) $$\frac{R_{e,true}-A}{\varepsilon_{e,pl}^{n}}=\frac{R_{m,true}-A}{\varepsilon_{m,pl}^{n}}=B\;$$

Consequently:

(12) $$n=\ln\left(\frac{R_{e,true}-A}{R_{m,true}-A}\right)/\ln\left(\frac{\varepsilon_{e,pl}}{\varepsilon_{m,pl}}\right)$$

The C coefficient is determined from the second term of Equation (9) for a given strain rate (Table 3).

(13) $$C=\left(\frac{R_{m,true}\left(\dot{\varepsilon }\right)}{R_{m,true}\left(\dot{\varepsilon_{0}}\right)}-1\right)/\ln\left(\frac{\dot{\varepsilon }}{{\dot{\varepsilon }}_{0}}\right)\;$$

The actual values of the strength limit are determined from Formula (6) (Table 3).

(14) $$R_{m,true}=R_{m}\left(1+\varepsilon_{nom}\right)$$

**Figure 3:**
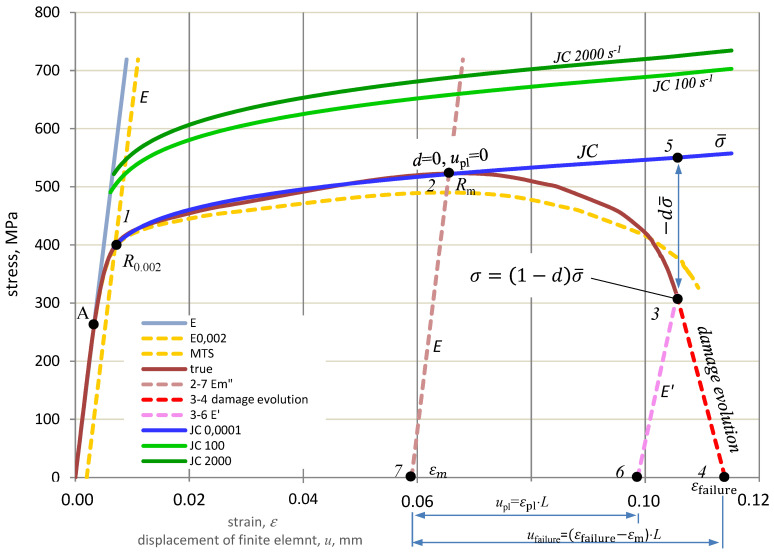
Failure diagram of the true characteristics of Al7.5Mg (*σ_true_*− *ε_true_*).

The average value was assumed constant C = 0.018893. The characteristics of JC for selected strain rates are shown in Figure 3.

The values for the temperature component can be taken based on the literature [21,26,27]. They are similar for most steels, so:

- Ambient temperature *θ*_0_ = 293.15 K;
- Melting temperature *θ*_top_ = 850 ÷ 855 K;
- Thermal coefficient *m* = 1.3 ÷ 1.7.

## 3. The Failure Mechanism for AL7.5Mg

The material failure model used in CAE programs is detailed in the works [21,28,29,30,31]. The value of the destructive deformation is a function of the so-called stress state indicator *η_TRIAX_* (stress triaxiality). It is the ratio of the pressure being the mean of the principal stresses to the Huber–Mises–Hencky (HMH) reduced stress *σ_HMH_* [28,29,31].

(15) $$\eta_{\mathrm{TRIAX}}=\frac{p}{\sigma_{\mathrm{HMH}}}$$

where:

$$p=\frac{1}{3}\left(\sigma_{x}+\sigma_{y}+\sigma_{z}\right)$$

$$\sigma_{\mathrm{HMH}}=\frac{1}{\sqrt{2}}\sqrt{{\left(\sigma_{x}-\sigma_{y}\right)}^{2}+{\left(\sigma_{y}-\sigma_{z}\right)}^{2}+{\left(\sigma_{z}-\sigma_{x}\right)}^{2}+6\left(\tau_{xy}{}^{2}+\tau_{zy}{}^{2}+\tau_{xz}{}^{2}\right)}$$

The triaxiality coefficient is an excellent identifier for the state of stress in complex states where it is difficult to discern whether an element is in compression, tension, bending, or twisting. For the uniaxial stretching state, the value of the triaxiality coefficient is equal to 0.33 (Table 4).

The failure mechanism for AL7.5Mg is shown in Figure 3, where 0–1 is the elastic range, 1–2 is the plastic range (hardening), and 2 is the initiation of the destruction process. Above 2, if the material model has no failure criteria, the stresses will vary to point 5 and beyond. If the load in point 2 disappears, the deformation will drop to point 7 along path 2–7 parallel to path 0–1. In the failure model, point 5 is above point 3 in curves 2–4. This is where strength (softening) is lost. The 2–4 degradation or failure curve is defined by parameter *d*, which takes values from 0 to 1. The stress on the degradation curve is:

(16) $$\sigma =\left(1-d\right)\bar{\sigma }$$

The material fracture occurs in point 4 after reaching the value of the fracture deformation $\varepsilon_{\mathrm{failure}}^{pl}$. However, if the element breaks or the forces loading the element disappear, e.g., in point 3 during the degradation of the material on the curve 2–4, then the remaining elastic forces will reduce its deformation to point 6 along the 3–6 path, which is not parallel to the 0–1 path. The evolution of failure determines the degree of degradation at which material failure will occur. The value *d* = 0 means that the plastic stress has reached the value of *R_m_*, but the material has not yet been degraded, while the value *d* = 1 means complete degradation of the material. The failure evolution is described as a function of the plastic displacement of the *u_pl_*, defined as [33]:

(17) $$u_{pl}=L\cdot \varepsilon_{pl}\;$$

where *L* is the characteristic length of the FEM element.

The rate of evolution of failure describes the path along which material degradation develops. In CAE programs, linear, exponential, and tabular descriptions are adopted. The linear relationship is expressed as the ratio of plastic displacement to failure displacement:

(18) $$d=\frac{u_{pl}}{u_{\mathrm{failure}}}$$

Table 5 lists the points from the diagram in Figure 3, based on which the failure parameters for tensile strength of Al7.5Mg were determined.

Following these parameters, calculations were carried out for uniaxial stretching:

$$\varepsilon_{\mathrm{failure}}=\varepsilon_{4}-\varepsilon_{7}=0.114-0.059=0.055\;d\bar{\sigma }=\sigma_{5}-\sigma_{3}=550.1-313.3=346.1\;\mathrm{MPa}\;\mathrm{since}\;\sigma =\left(1-d\right)\bar{\sigma }\mathrm{so}\;\;d=1-\frac{\sigma }{\bar{\sigma }}=1-\frac{313.3}{550.1}=0.4304\;E^{\prime }=\left(1-d\right)E=\left(1-0.4304\right)\cdot 79.9=45.5\;\mathrm{Gpa}\;u_{\mathrm{failure}}=0.055\cdot \;L$$

Summarizing the tested Al7.5Mg can be described by the following equations:

Young’s modulus: E=79.9 GPa

Johnson–Cook Model:

$$\sigma =\left(258+448.7\cdot \varepsilon^{0.1877}\right)\left[1+0.01889\;\cdot \ln\left(\frac{\dot{\varepsilon }}{0.0001}\right)\right]\left[1-{\left(\frac{\theta -293.15\;}{855}\right)}^{1.3}\right]$$

Failure parameters:

d=0.4304; $\varepsilon_{\mathrm{failure}}=0.055;\;\eta_{Triax}=0.33$.

## 4. Material Data of the Elements of the Aircraft Structure

Rivets are the main research object in this work. For this reason, simplified bilinear material models were adopted for the remaining elements of the aircraft structure. The plating and reinforcement profile are made of the aluminium alloy 7075-T6 with the following mechanical properties:

- Young’s modulus *E* = 71.7 GPa;
- Yield stress *R*_0.002_ = 463 MPa;
- Strength limit *R_m_* = 530 MPa;
- Poisson number *ν* = 0.33; and
- Density *ρ* = 2810 kg/m^3^.

The material of the hammer and support is made of high-strength steel with the following mechanical properties:

- Young’s modulus *E* = 2.09 × 10^5^ MPa;
- Yield stress *R_e_* = 665 MPa;
- Strength limit *R_m_* = 776 MPa;
- Poisson number *ν* = 0.33; and
- Density *ρ* = 7850 kg/m^3^.

## 5. FEM Simulation of the Impact Strength of Riveted Aircraft Structure

The obtained model of the AL7.5Mg alloy should be verified. For this purpose, we performed a FEM simulation of the strength of the riveted aircraft structure, loaded with the impact of the drop hammer. The results of the FEM simulation were compared with the results of the same experiment.

### 5.1. Research Object, Its Geometry and Discretisation

**Figure 4:**
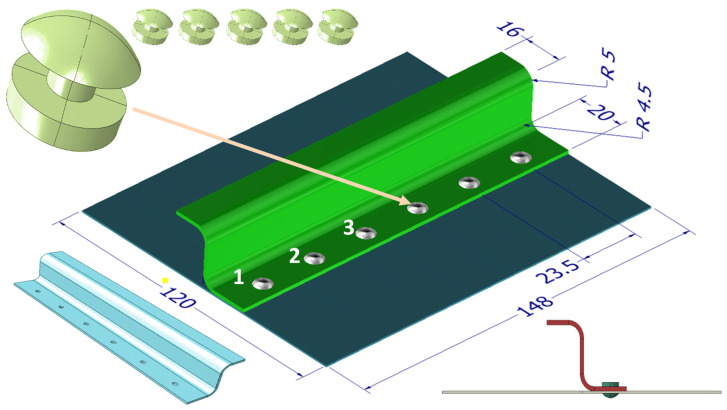
The geometry of the test object, the numbering of rivets [34].

The test object is a fragment of the aircraft plating made of the 7075-T6 aluminium alloy with dimensions of 120 × 148 × 0.8 mm. It is connected with six rivets with a diameter of 3 mm with an S-shaped reinforcing profile with dimensions of 16 × 25 × 20 × 1.6 mm. The rivets are symmetrically spaced every 23.5 mm. The aluminium alloy Al7.5Mg after the hydro-extrusion process was proposed as a material for rivets. The geometry of these elements and the basic dimensions are shown in Figure 4. A fragment of the aircraft structure will be subjected to the impact of the drop hammer.

**Figure 5:**
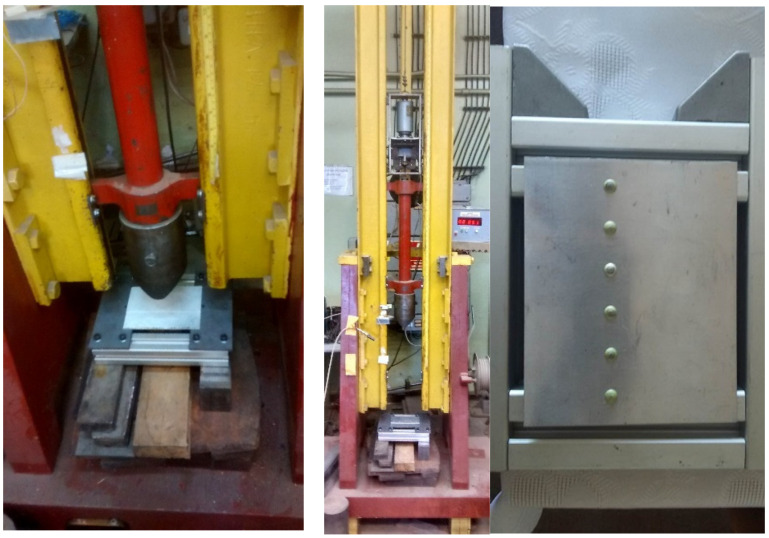
The course of the experiment on the drop hammer at the Polish Naval Academy. From left: drop hammer station in the laboratory; view of the bumper and aircraft structure; view of the aircraft structure from the impact side; bumper at the moment of impact.

In the Fundamentals of Technology Laboratory of the Naval Academy (AMW) in Gdynia (Poland), an experiment was conducted on an identical object loaded with a drop bumper. The aircraft structure was hit by a 35.81 kg bumper falling from a height of 30 cm to its flat side (Figure 5). At the moment of impact (contact), the bumper reached a speed of 2.42 m/s.

**Figure 6:**
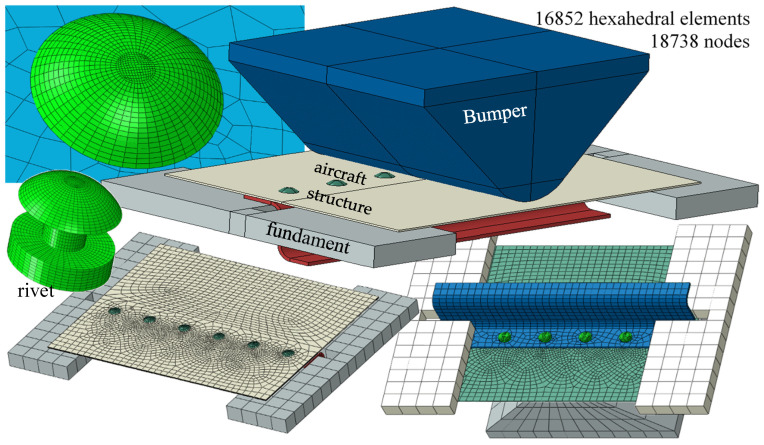
Geometry and discretisation of aircraft structure elements in the CAE program.

The geometry of individual elements of the aircraft structure was reflected in the CAD program, where it was also assembled into a set (Figure 6). It was exported to the CAE program, where steel supports and a bumper were reflected. The entire structure with supports and a hammer was discretised by 124,643 linear eight-node hexagonal elements delimited in space by 140,899 nodes, giving 845,394 degrees of freedom (Figure 6). Each rivet was divided into 16,852 elements delimited in space by 18,738 nodes. The size of the rivet mesh is *L* = 0.2 mm.

### 5.2. Boundary Conditions and Loads

**Figure 7:**
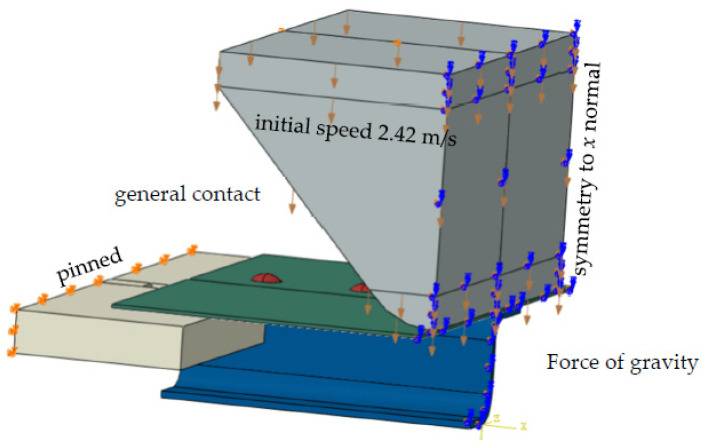
Boundary conditions and loads.

The following boundary conditions were assumed: all displacements were suspended on opposite surfaces with a rectangular base on which the sample rests. The hammer was given an initial velocity at the moment of impact of 2.42 m/s and made to ensure vertical movement. In addition, all elements were mass-loaded with the acceleration due to gravity of 9.81 m/s, which is a gravitational load. All individual parts were given “general contact” interactions in the assembly, i.e., mutual interactions between all elements. The symmetry of the task was used, and half of the structure was solved. Displacements in the perpendicular direction and rotations in planes perpendicular to the plane of symmetry were obtained from all nodes lying on the plane of symmetry (Figure 7).

### 5.3. Equation of Motion

Due to the impact nature of the load, material nonlinearities, and interactions between structural elements, the FEM dynamic equation of motion (dynamic explicit analysis) was solved using the Newmark numerical integration method in the form of:

(19) $$M\left(U\right)\ddot{U}+C\dot{U}+K\left(U,\dot{\varepsilon },\varepsilon_{pl},\varepsilon_{\mathrm{failure}}\right)U=F\left(v\left(t\right),m,\;BC,C_{int},t\right)+G\;U\left(t_{0}\right)=U_{0}\;\dot{U}\left(t_{0}\right)={\dot{U}}_{0}$$

where:

***K***—structure stiffness matrix;

***M***—inertia matrix;

C=αM+βK—damping matrix, where *α* and *β* are constant coefficients;

$U,\dot{\;U,}\ddot{\;U}$—vector of displacement, velocity and acceleration;

$U_{0}$, ${\dot{U}}_{0}$—initial conditions, displacements and velocities;

***F***—vector of loads;

$\dot{\varepsilon }$—strain rate;

$\varepsilon_{pl}$—vector of plastic strains (JC model);

$\varepsilon_{\mathrm{failure}}$—failure parameters f(d,$\;\eta_{Triax})$;

v(t)—hammer speed;

m—the mass of the bumper;

*BC*—the influence of boundary conditions;

*C_int_*—interactions and contact forces between colliding structural elements; and

***G***—the force of gravity

### 5.4. The Time Step

One of the problems in the numerical integration of motion equations is selecting the appropriate time step, which depends on the structure elements’ elasticity modulus and material density [35]. The time step value is the ratio of the smallest size in single-element mesh to the speed of the elastic (acoustic) wave propagation in the element material, i.e.,

(20) $$\Delta t=\frac{h}{a}$$

where:

*h*—the smallest size of single element mesh; and

a=Eρ—elastic (acoustic) wave velocity.

For aluminium, the speed of sound is approximately 5100 m/s. In the aircraft structure, the smallest size in the mesh of finite elements has rivets *h* = 0.2 mm, so the required time step must be less than 3.92 × 10^−^^8^ s.

## 6. FEM Simulation Results—Rivets Made of Al7.5Mg Alloy

**Figure 8:**
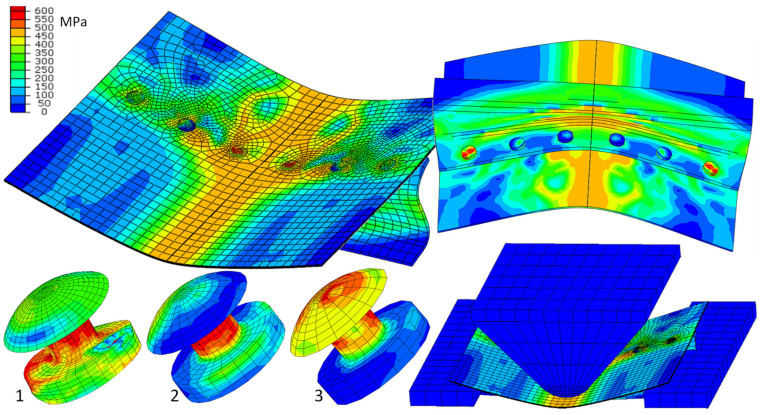
Reduced stress HMH in the elements of aircraft structures, *t* = 20 ms.

**Figure 9:**
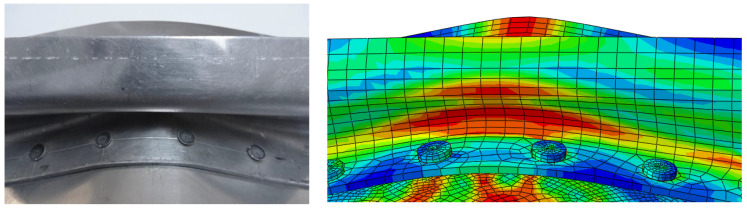
State of deformation after an impact, experiment, and FEM simulation.

**Figure 10:**
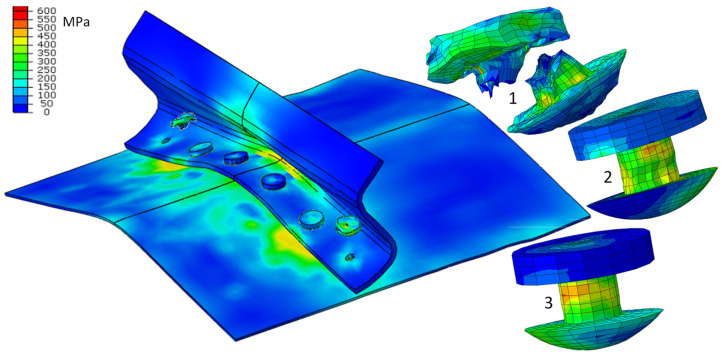
Reduced stress HMH in the elements of aircraft structures after impact; *t* = 90 ms.

Only selected results are presented, mainly the HMH reduced stresses (Figure 8). The focus is on stresses in rivets, as they are of extreme value. The stresses of the HMH exceed the limits of their strength and break as a result. The form of deformation is similar to the results of the experiment (Figure 9). Figure 10 shows the shape of the aircraft structure after impact and in rivets.

**Figure 11:**
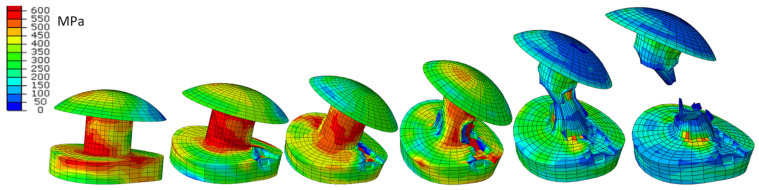
Deformation and distribution of HMH (MPa) reduced stresses in extreme rivets; *t* = 5, 10, 20, 30, 40, 48 ms.

**Figure 12:**
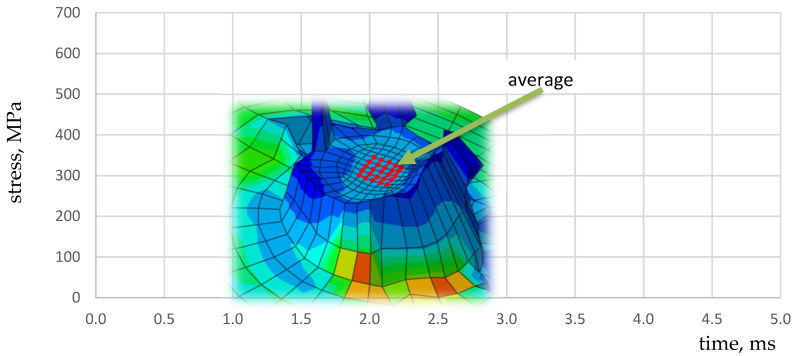
HMH reduced stress distribution in the rivet; the red line indicates average value of the internal nodes is 25.

Figure 11 and Figure 12 show the development of deformation and the HMH reduced stress state in the extreme rivets.

**Figure 13:**
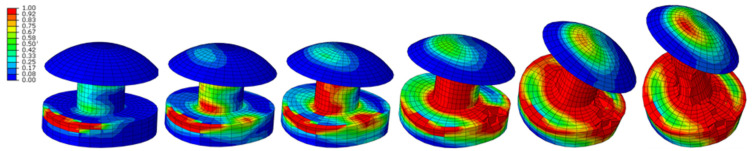
Development of destruction in the elements of the extreme rivets; *t* = 3, 5, 6, 8, 20, 39 ms.

Figure 13 shows the failure criterion in extreme rivets at selected time moments. Already in 3 ms, it reaches the value of 1 in several finite elements and develops into the following elements, covering the entire rivet core in 20 ms.

**Figure 14:**
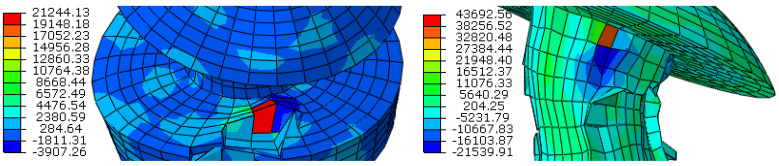
The strain rate in the extreme elements of rivets; *t* = 10 and 38 ms.

In the rivet elements, the average strain rates oscillate within the limits of 0–2000 s^−1^, temporarily reaching the values of 5000 s^−1^, while locally, in the broken elements, the strain rates reach a value of up to 43,700 s^−1^ (Figure 14).

## 7. Discussion

The simulation results for a fragment of the aircraft structure were compared with the experiment results. Satisfying compliance of the deformation state after impact was obtained. This confirms the correctness of the modelled task and description of the tested material, the AL7.5Mg alloy after hydro-extrusion.

The extreme rivets were broken, but it should be noted that a fragment of the structure was analysed. The rivets will probably not fail in the continuous plating, with an impact of similar parameters, but the structure is likely to become unsealed. The internal rivets withstand the set load due to aluminium’s relatively high strength limit and high plasticity. The static strength limit is 523 MPa, and at deformation rates over 500 s^−1^, it increases over 670 MPa. With rivets of larger diameter, they will not break.

In this paper, the material characteristics of the tested material were developed for uniaxial stretching. The triaxiality coefficient identifies the plastic failure depending on the stress state, the value of which is 1/3, and for uniaxial compression, the value is 1/3. Other values of the triaxiality factor can be obtained by stretching the sample with a notch. The shape of the notch changes the direction of the forces, which was shown in the works [36,37,38], which investigated samples with a notch. In these studies, the triaxiality coefficient determined by the Bridgman equation [15] was used to identify the direction of the forces:

(21) $$\eta_{\mathrm{TRIAX}}=\frac{1}{3}+\ln\left(\frac{r}{2R}+1\right)$$

where (Figure 15):

*r*—radius of the smallest cross-sectional area, mm; and

*R*—notch radius, mm.

The tests of the Al7.5Mg aluminium alloy will be continued in order to complete the material characteristics for the remaining values of the triaxiality coefficient, which will be the subject of the next article.

The characteristics of the Al7.5Mg alloy presented here and verified can be used to simulate much more complex structures and objects, which requires appropriate computing power. The presented task was calculated on an 8-core PC (i7—2.80 GHz) and lasted about 20 h.

## 8. Conclusions

Strength tests carried out in the laboratory allowed us to determine the mechanical properties of the AL7.5Mg alloy after hydro-extrusion in the deformation speed range of 0–2000 s^−1^. The following results were obtained: yield strength *R_e_* = 395.3 MPa; strength limit *R_m_* = 523.1 MPa at deformation 0.067; Young’s modulus *E* = 7.9 × 10^4^ MPa. The AL7.5Mg alloy after hydro-extrusion has favourable plastic and strength properties.

Based on the above research, a constitutive model of Johnson–Cook AL7.5Mg was developed, which is ready for use in CAE programs.

The increase in the strain rate above 2000 s^−1^ increases the strength of the tested alloy to 690 MPa.

The failure parameters for tensile strength (*η* = 0.33) were determined. The same parameters were adopted for compression for *η* = −0.33. Failure parameters: d=0.4304; $\varepsilon_{\mathrm{failure}}=0.055$. For the remaining values of the *η* coefficient, tensile tests of the notched samples should be carried out using the Bridgman equation [15].

The use of rivets made of Al7.5Mg alloys subjected to SPD processes may contribute to the reduction in the cross-sectional area of the joints of aircraft structures. This will reduce the weight of the aircraft.

The presented characteristics constitute a ready-made solution to be implemented in CAE programs. They do not deal with the influence of machining on mechanical properties, but represent an engineering solution for implementation in calculations related to the use of Al7.5Mg alloys.

The correctness of the task can be verified only by an actual experiment. When designing individual objects, e.g., ships and vehicles, so-called crash tests are impossible or too expensive in many cases. FEM simulations are the only source of information about the behaviour of the structure under dynamic loads.

## Figures and Tables

**Figure 15:**
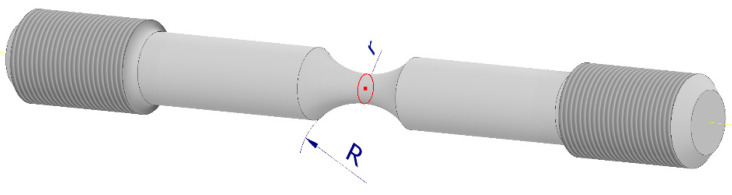
Notched specimen used in Bridgman’s work.

**Table 1:** Calculation of true and plastic material characteristics.

*Ε_nom_* Nominal Strain -	*σ_nom_* Nominal Stress Mpa	*ε_true_* True Strain -	*ε_pl_* Plastic Strain -	*σ_true_* True Stress MPa
(3)	(4)	(5)	(6)	(7)
$\varepsilon_{nom}=\frac{\Delta l}{l_{0}}$	$\sigma_{nom}=\frac{F}{A_{0}}$	$\varepsilon_{true}=\ln\left(1+\varepsilon_{nom}\right)$	$\varepsilon_{pl}=\varepsilon_{true}-\frac{\sigma_{true}}{E}$	$\sigma_{true}=\sigma_{nom}\left(1+\varepsilon_{nom}\right)$
0.00032	27.3	0.00032	0	27.3
0.00218	177.8	0.00217	0	178.2
0.00285	229.4	0.00285	0	230.0
*ε_H_* = 0.0032	*σ_H_* = 258.0	0.00320	0.00001	258.8
0.00355	276.7	0.00354	0.00007	277.7
0.00431	319.1	0.00430	0.00029	320.4
0.00617	382.0	0.00616	0.00134	384.4
*ε_e_* = 0.0068	*R_e_* = 392.6	*ε_e.true_* = 0.00675	*ε_e.pl_* = 0.00181	*R_e.true_* = 395.3
0.00746	401.5	0.00743	0.00237	404.5
0.01266	429.1	0.01258	0.00714	434.5
0.03251	462.2	0.03199	0.02602	477.2
0.05680	487.7	0.05525	0.04880	515.4
*ε_m_* = 0.0653	*R_m_* = 490.0	0.06330	0.05677	522.1
0.06678	489.9	0.06465	0.05811	522.6
0.06929	489.2	*ε_m.true_* = 0.0670	*ε_m.pl_* = 0.0605	*R_m.true_* = 523.1
0.07451	485.7	0.07187	0.06533	521.9
0.10670	364.4	0.10139	0.09633	403.3
*ε_f_* = 0.1103	*R_f_* = 305.9	*ε_f.true_* = 0.1055	*ε_f.pl_* = 0.1016	*R_f.true_* = 313.3

**Table 2:** Summary of test results on a rotary hammer.

Sample Name	*φ*	Measuring Length	Area *A*_0_	Breaking Force *F_m_*	Hammer Rotational Speed	Strain Rate	Dynamic Ultimate Strength *R_m_*
	mm	mm	mm^2^	kN	m/s	s^−1^	MPa
Al7.5Mg_v1	5.05	18.69	20.03	12.65	10	535	631.6
Al7.5Mg_v2	5.04	19.36	19.95	12.80	20	1033	641.6
Al7.5Mg_v3	5.08	19.33	20.27	13.08	30	1552	645.3
Al7.5Mg_v4	5.06	18.53	20.11	13.07	40	2159	650.0

**Table 3:** Ultimate strength R*_m,nom_*, $R_{m,true,\dot{\varepsilon }}$ , $R_{m,JC,\dot{\varepsilon }}$, C for various strain rates.

Strain Rate	${\dot{\varepsilon }}_{0}=0.0001\;s^{-1}$	$\dot{\varepsilon }=535\;s^{-1}$	$\dot{\varepsilon }=1033\;s^{-1}$	$\dot{\varepsilon }=1552\;s^{-1}$	$\dot{\varepsilon }=2159\;s^{-1}$
$R_{m,nom,\dot{\varepsilon }}$, MPa	490	631	641	645	649
$R_{m,true,\dot{\varepsilon }}$, MPa	523	672	683	687	692
$R_{m,JC,\dot{\varepsilon }}$, MPa	523	676	682	686	689
C	-	0.01847	0.01898	0.01897	0.01916

**Table 4:** The values of the triaxiality coefficient for selected 3D cases [32].

	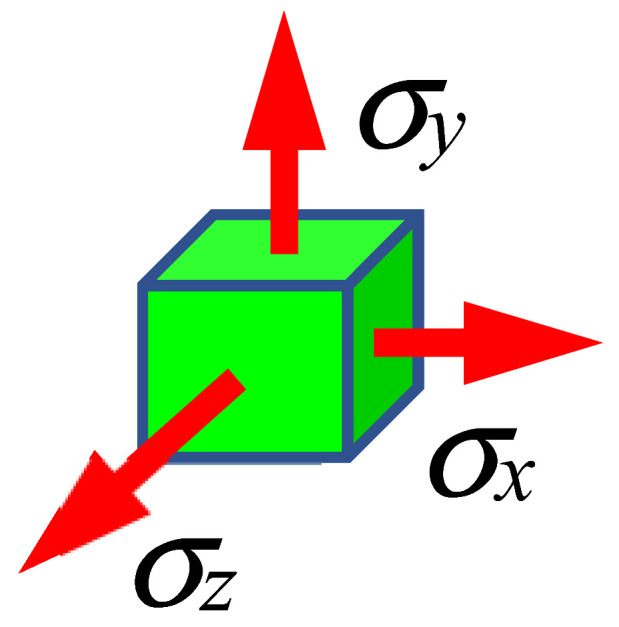	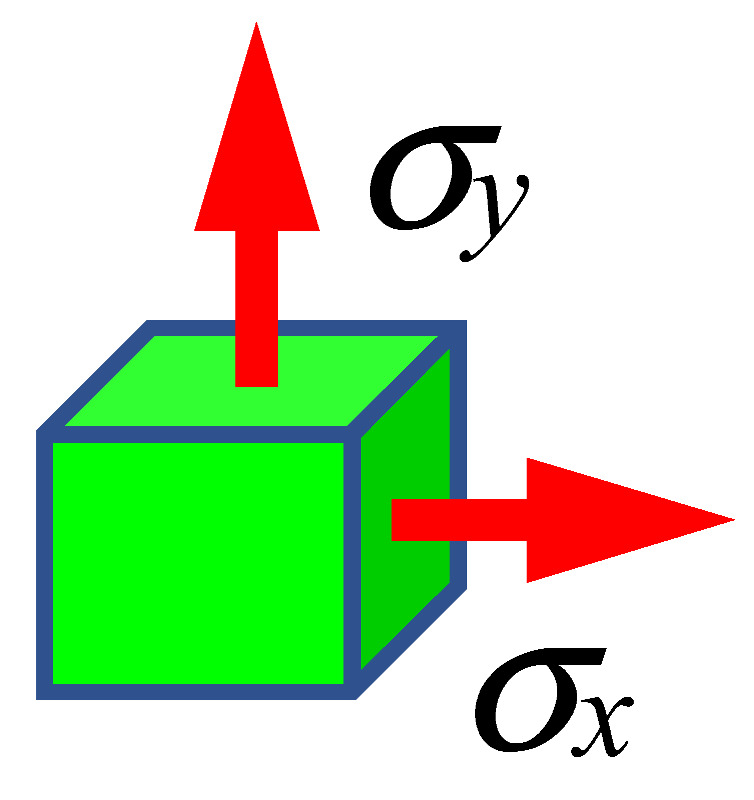	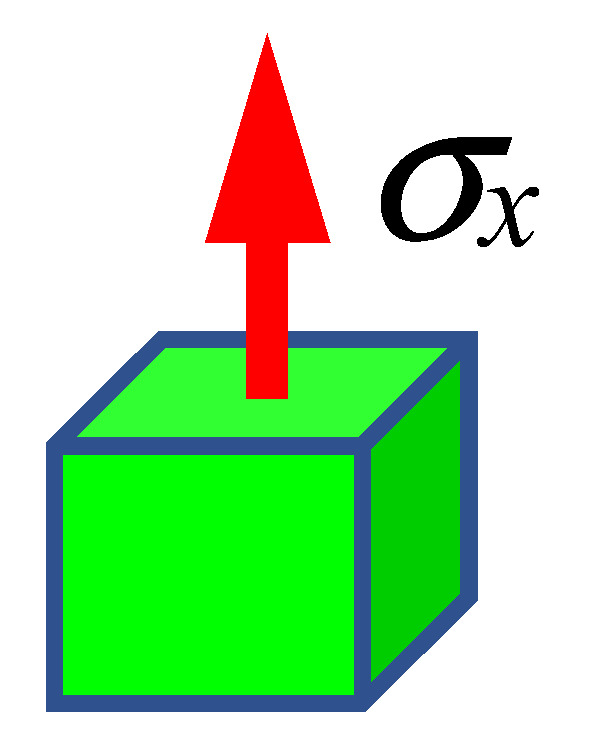	$p=\frac{1}{3}\sigma_{x}\;\;\;\;\;\;\;\sigma_{y}=\sigma_{z}=0$ $\sigma_{\mathrm{HMH}}=\frac{1}{\sqrt{2}}\sqrt{\sigma_{x}{}^{2}+\sigma_{x}{}^{2}}=\sigma_{x}$ $\eta_{\mathrm{TRIAX}}=\frac{\sigma_{x}}{3\sigma_{x}}=\frac{1}{3}$
Principal stresses	*σ_x_* = *σ_y_* = *σ_z_* > 0	*σ_x_* = *σ_y_* *σ_z_* = 0	*σ_x_* > 0 *σ_y_* = *σ_z_* = 0	
*η_TRIAX_*	∞	0.66	0.33	

**Table 5:** The values in Figure 3 used in the calculations.

Point Label	Strain	Stress	Remarks
	$\varepsilon_{el},\;-$	$\sigma_{true},\;\mathrm{MPa}$	
*A*	0.0040	258.0	$\mathrm{Elastic}\;\mathrm{range}\;\mathrm{of}\;\mathrm{the}\;\mathrm{material}\;\sigma_{H}=\sigma_{pl=0}$
1	0.0068	395.3	Yield point *R_e_*
2	0.0671	523.1	Ultimate tensile strength *R_m_*
3	0.1055	313.3	Sample fracture
4	0.1140	0.00	*d* = 1 material total degradation
5	0.1055	550.1	Stresses in the material model without failure parameters
6	0.0987	0.00	Fracture deformation
7	0.0590	0.00	Deformation at ultimate strength *R_m_*, *d* = 0

## Data Availability

Not applicable.
